# Vitamin D receptor gene polymorphisms and susceptibility to urolithiasis: a meta-regression and meta-analysis

**DOI:** 10.1186/s12882-020-01919-1

**Published:** 2020-07-10

**Authors:** Danyal Imani, Bahman Razi, Arezou Khosrojerdi, Kaivan Lorian, Morteza Motallebnezhad, Ramazan Rezaei, Saeed Aslani

**Affiliations:** 1 Department of Immunology, School of Public Health, Tehran University of Medical Sciences, Tehran, Iran; 2 Department of Hematology, School of Medicine, Tarbiat Modares University, Tehran, Iran; 3 Department of Medical Immunology, Faculty of Medical Sciences, Tarbiat Modares University, Tehran, Iran; 4 Department of Physiology, School of Medicine, Tehran University of Medical Sciences, Tehran, Iran; 5 Department of Immunology, Faculty of Medicine, Iran University of Medical Sciences, Tehran, Iran; 6 Immunology Research Center, Iran University of Medical Sciences, Tehran, Iran; 7 Department of Immunology, Medical School, Shahid Beheshti University of Medical Sciences, Tehran, Iran; 8 Department of Immunology, School of Medicine, Tehran University of Medical Sciences, Tehran, Iran

**Keywords:** Vitamin D receptor, Urolithiasis, Meta-analysis, Polymorphism

## Abstract

**Background:**

The currently available data with respect to the association between *vitamin D receptor* (*VDR*) gene polymorphism and risk to urolithiasis are inconclusive and inconsistent. Hence, an exhaustive meta-analysis can solve the discrepancies and provide a hint for upcoming investigations. Herein, a meta-analysis was carried out to attain a conclusive estimate of the association between *VDR* gene single nucleotide polymorphisms (SNPs) and urolithiasis risk.

**Methods:**

The major databases, including ISI Web of science, Scopus, and PubMed/MEDLINE were searched systematically from until June 2020 to retrieve all relevant studies. Association between *VDR* gene polymorphisms, including FokI (rs2228570), TaqI (rs731236), BsmI (rs1544410), and ApaI (rs7975232), and urolithiasis risk was evaluated using pooled odds ratio (OR) and their corresponding 95% confidence interval (CI). Additionally, to seek for the potential source of heterogeneity, meta-regression analyses were exerted.

**Results:**

Literature search led to finally finding of 33 studies evaluating the *VDR* gene SNPs and urolithiasis risk. It was observed that none of the four SNPs were significantly associated with urolithiasis predisposition. However, subgroup analysis confirmed higher risk of urolithiasis in East-Asian and Caucasian population with ApaI and *TaqI* gene polymorphism. The analyses of sensitivity acknowledged the results stability.

**Conclusion:**

Although this meta-analysis did not support the association of *FokI, TaqI, BsmI*, and *ApaI* in the overall polled analysis, it suggests that *ApaI* and *TaqI* SNPs is associated with increased risk of urolithiasis in East-Asian and Caucasians populations.

## Background

Urolithiasis is known as one of the prevalent diseases among urological disorders that has been associated with many complicated factors [1]. Urolithiasis is characterized by a high recurrence incidence, and its prevalence rate is 4–20% in developed countries, and the disease incidence continues to increase [2]. It is a multifactorial disorder, resulting from environmental influences, metabolic defects and genetic factors [3]. Numerous investigations recognized the importance of genes in this disorder.

Studies have shown that several genetic factors including single nucleotide polymorphisms (SNPs) in osteopontin (OPN), progestin and adiporeceptor 6 (PAQR6), calcium-sensing receptor (CaSR), and vitamin D receptor (VDR) are correlated with the risk of urinary calcium stone formation [4–7]. In spite of attribution of a genetic background in susceptibility to urolithiasis, little has been identified with respect to the relevant genetic loci for the disease. Two genome-wide association studies (GWASs) recognized four risk susceptibility genes, including *CLDN14* in Europeans and Japanese [8, 9], *INMT-FAM188B-AQP1*, *RGS14-SLC34A1-PFN3-F12*, and *DGKH* in Japanese [9]. That notwithstanding, these studies suggested further studies was needed to identify more risk loci as well as to recognize the molecular mechanisms attributed to the urinary calculi.

Broadly speaking, complex interactions of genetics and environmental factors, such as water intake, diet, urine pH, and infections have been associated with the etiopathogenesis of urolithiasis [10]. The underlying mechanisms of the development of calcium-containing stones, which are the most common type of kidney and bladder stones, have not fully been divulged [11]. Nowadays, the possibility of both free and fixed stones development has been suggested. The widely accepted explanation of the development of such stones relies on the increased solubility of the lithogenic elements in the urine [11]. Furthermore, it has been contemplated that the deposition of initial crystals occurs in the lumens of renal tubules [12, 13]. However, recent observations imply that a development of Randall plaques in the renal papilla is the initial trigger of stone formation [14]. Such plaques are developed when calcium phosphate crystals are deposited in the basement of the thin loops of Henle and then extend into the urothelium. Calcium oxalate stones, which are responsible for almost 80% of all urinary stones, are developed after formation of calcium phosphate crystals. In fact, the binding of more calcium oxalate as well as matrix molecules present in the urine to the Randall plaques accelerates the formation of calcium oxalate stones [15].

Recent studies have demonstrated that receiving vitamin D supplements maybe put the individual at risk of developing kidney stones disease [16]. Moreover, vitamin D has an important role in calcium metabolism, such as absorption of calcium from intestine and its reabsorption in the kidneys. it through increasing the serum calcium levels could enhance the risk of urinary stone formation [17]. Vitamin D functions are dependent on the expression and nuclear activation of VDR [18]. Therefore, any alteration in the VDR may change the calcium metabolism, thus alter the urolithiasis risk. Taken together, studies have recommended that VDR play an essential role in the pathogenesis of urolithiasis [19].

The human *VDR* gene is placed on the chromosome 12q12–q14 that harbors more than 200 SNPs, among which FokI (or rs2228570), TaqI (or rs731236), BsmI (or rs1544410), and ApaI (or rs7975232) polymorphisms have been extensively investigated. *VDR* gene has at least five promoter regions, six untranslated exons, and eight protein-coding exons, which are alternatively spliced into BsmI, FokI, ApaI, and TaqI [20].. BsmI and ApaI are placed on the 9th intron of the 3′ terminal, TaqI is located on the 9th exon of the 3′ terminal, and FokI is established on the promoter of the 5′ terminal. Studies have reported that BsmI and TaqI SNPs are not involved in altering the protein structure of VDR; however, they have been suggested to play a role in the translation efficiency and stability of the corresponding mRNA [21]. Numerous studies have indicated the association of polymorphisms in the *VDR* gene with several human diseases [22, 23].

A series of studies investigated the association between these polymorphisms of *VDR* gene and the risk of urolithiasis, but the findings have been conflicting [24–50]. The inconsistent results were possibly because of clinical heterogeneity, small sample sizes, and low statistical power. In addition, previous meta-analyses [51–53] appeared to be out of date due to the availability of new data [45–50]. Therefore, we performed the most up to date meta-analysis with the aim of obtaining more accurate and updated results.

## Methods

This study was performed in a stepwise process in accordance with the guidelines of the 2009 Preferred Reporting Items for Systematic Reviews and Meta-analyses (PRISMA) statement [54]. Besides, the current project does not contain any studies with human participants or animals performed by any of the authors. Registration in the International Prospective Register of Systematic Reviews (PROSPERO) was carried out.

### Literature identification

A detailed systematic search was performed to identify candidate studies evaluating the associations between *VDR* gene polymorphisms and urolithiasis susceptibility (prior to June 2020). Three electronic databases, including Web of Science, MEDLINE, and Scopus were searched and for all of them, following combination of key words were used: (“urolithiasis” or “Kidney stone disease”) AND (“VDR” OR “vitamin D receptor”) AND (“polymorphisms” OR “SNP” OR “variation” OR “mutation”). Cross references within both original and review publications were done for additional pertinent studies. Original data were collected from English language and human population studies.

### Inclusion/exclusion criteria

Studies included in quantitative analysis if met the following inclusion criteria: a) studies concerning the association between *VDR* gene polymorphisms and urolithiasis risk; b) Studies with case-control design; c) studies reporting sufficient data of genotype or allele frequency in order to calculate odds ratios (ORs) and 95% confidence intervals (CIs). On the other hand, duplicate data, case report, book chapter, review, letter, and abstracts were excluded.

### Data extraction

All required data were extracted conforming to the standardized extraction checklist for the following data: the first author’s name, journal and year of publication, country of origin, ethnicity, number of subjects in the case and control groups, mean or range of age, genotyping method, genotype counts in the case and control group. The extracted items were compared and any possible discrepancies were resolved by consensus.

### Quality assessment

Methodological quality of eligible studies was evaluated by Newcastle–Ottawa Scale (NOS), a validated scale for non-randomized studies in meta-analysis. This scale consists of 3 parts with a total of 9 items. In this regards, studies with scores 0–3, 4–6 or 7–9 were of low, moderate, or high-quality, respectively [55].

### Statistical analysis

For evaluating the distribution of the genotype frequencies to see if it is deviated from Hardy–Weinberg equilibrium (HWE) in the control group, the χ2-test was employed [56]. The quality of association between *VDR* gene SNPs and urolithiasis risk was evaluated by the pooled OR and its corresponding 95% CI. Five different comparison model for FokI, TaqI, BsmI, and ApaI SNPs were as follow**:** dominant model, recessive model, allelic model, homozygote contrast, and heterozygote contrast. Presence of heterogeneity between included studies was estimated by Cochran’s Q-statistic (*P* value< 0.10 was considered as statistically significant). Besides, to report quantitative heterogeneity, the I-squared (I2) test was used. The fixed-effected model was used if PQ statistic> 0.10 or I2 was< 50%; otherwise, the random-effected model was applied [57, 58]. We assessed the predefined sources of heterogeneity among included studies by subgroup analysis and meta-regression analysis based on year of population, and genotyping method. The stability of our results was measured by sensitivity analysis. Additionally, sensitivity analysis was conducted in the presence of heterogeneity. Moreover, Begg’s test, Egger’s regression test and visual examination of the funnel plot were applied to measure publication bias (*P* value< 0.05 was considered as statistically significant) [59]. The data analyses were carried out using STATA (version 14.0; Stata Corporation, College Station, TX) and SPSS (version 23.0; SPSS, Inc. Chicago, IL).

## Results

### Specifications of the included studies

**Fig. 1:**
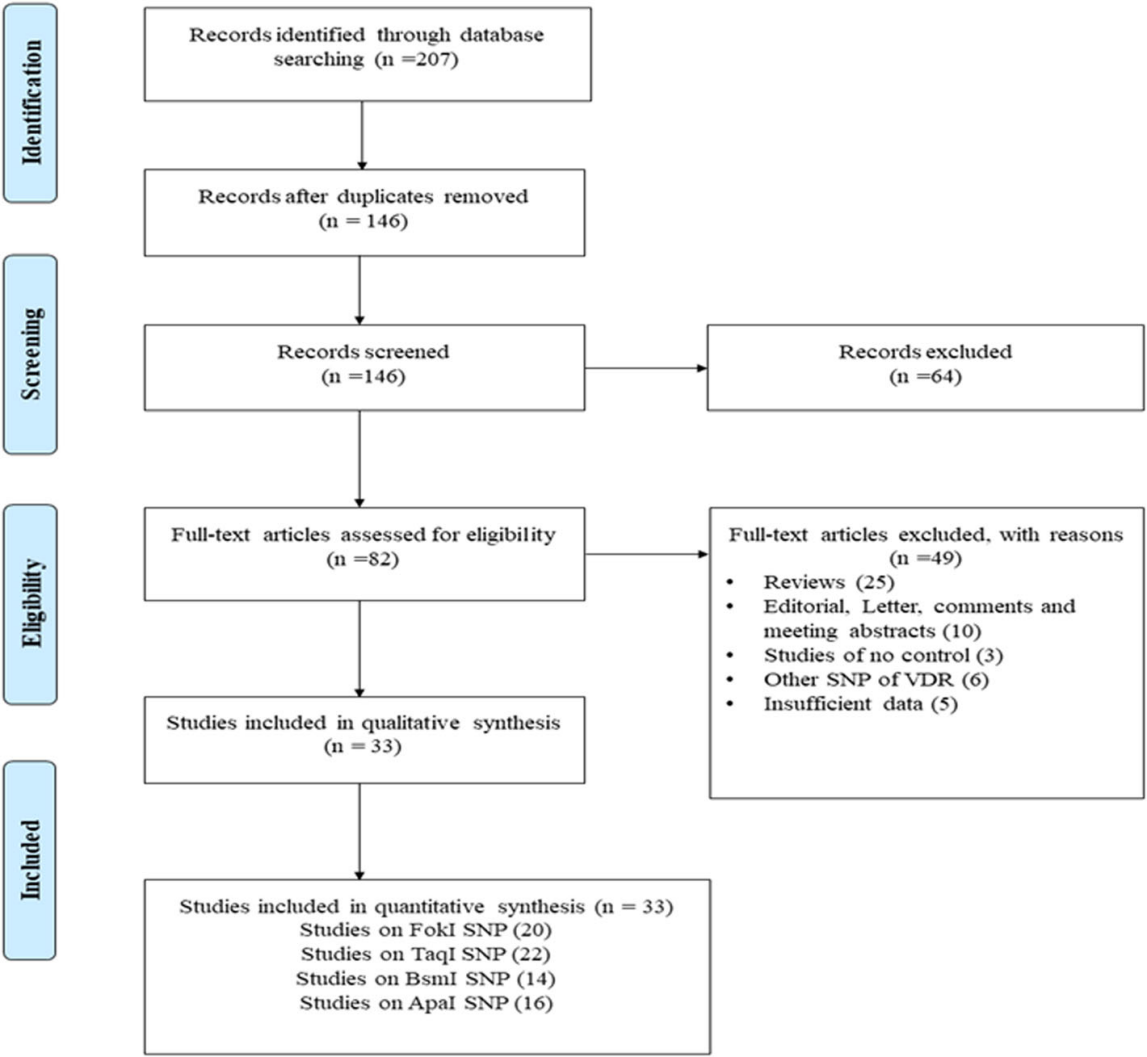
Flow diagram of study selection process

The exact process of literature searches and study selection is depicted in the Fig. 1. Early literature search eventuated in identification of 207 records, 33 of which met the final inclusion criteria and included in quantitative analysis. Among 33 eligible studies, 20 studies investigated the *FokI* SNP, 22 studies *TaqI* SNP, 14 studies *BsmI* SNP and 16 studies *ApaI* SNP. The studies were published between 1999 and 2020 and had an overall good methodological quality with NOS scores ranging from 5 to 8. Polymerase chain reaction-restriction fragment length polymorphism (PCR-RFLP) and Taq-Man were used by majority of included studies as genotyping method. Tables 1 and 2 summarized the characteristics of the included studies.

**Table 1:** Characteristics of studies included in meta-analysis of overall Urolithiasis

Study author	Year	Country	Ethnicity	Sex cases/controls	Total cases/controls	Age case/control (Mean)	Genotyping method	Quality score
**FokI (rs2228570)**								
Chen et al. (b)	2001	China	Asian	M = 101/42 F = 45/48	146 / 90	44.2 / 55.5	PCR–RFLP	6
Shaogang et al.	2003	China	Asian	M = 89/ 58 F = 61/22	150 / 80	43.6 ± 16/ 49 ± 19.6	PCR–RFLP	6
Rendina et al.	2004	Italy	European	M = 94/72 F = 65/52	159 / 124	43 ± 10.8 / 41.9 ± 10.4	PCR–RFLP	7
Mossetti et al.	2004	Italy	European	M = 66/ 73 F = 44/54	110 / 127	41.3 ± 13.5 / 41.06 ± 13.9	PCR–RFLP	6
Relan et al.	2004	India	Asian	M = 105/76 F = 45/24	150 / 100	39.3 ± 1.1 / 43.2 ± 2.05	PCR–RFLP	7
Bid et al. (a)	2005	India	Asian	M = NR F=NR	113 / 132	21–72 / 22–58	PCR–RFLP	6
Bid et al. (b)	2005	India	Asian	M = NR F=NR	50 / 60	2–14 / 4–16	PCR–RFLP	5
Liu et al.	2007	China	Asian	M = 161/159 F = 74/72	235 / 231	50.1 ± 12.3 / 51.7 ± 11.1	PCR–RFLP	8
Seo et al.	2009	Korea	Asian	M = 93/ 220 F = 185/313	278 / 533	49.9 / 40.1	PCR–RFLP	8
Mittal et al.	2010	India	Asian	M = NR F=NR	125 / 150	40 ± 11.5 / 41.5 ± 10.5	PCR–RFLP	7
Basiri et al.	2012	Iran	Asian	M = NR F=NR	102 / 106	43.4 ± 6.9 / 38.4 ± 6.9	SSP-PCR	6
Kaysar et al.	2012	China	Asian	M = NR F=NR	74 / 103	NR	PCR–RFLP	5
Wang et al.	2012	China	Asian	M = 279/263 F = 185/187	464 / 450	50.01 ± 10.19 / 50.45 ± 11.22	PCR–RFLP	8
Guha et al.	2015	India	Asian	M = 133 / 112 F = 67 / 78	200 /200	39.93 ± 11 / 38.13 ± 10	PCR	7
Cakir et al.	2016	Turkey	European	M = 65 / 52 F = 33 / 18	98 / 70	47.2 ± 16.3 / 42.6 ± 13.5	PCR–RFLP	6
Ergon et al.	2017	Turkey	European	M = NR F=NR	27 / 13	7.12 ± 2.64 / 6.92 ± 2.48	Tag-man	5
Subasi et al.	2017	Turkey	European	M = 26/22 F = 26/29	52 / 51	9.8 ± 3.5 / 10.3 ± 3.7	SNaPshot	5
Li et al.	2018	China	Asian	M = 100/60 F = 100/60	200 / 120	35.88 ± 14.2 / 36.16 ± 15.20	PCR	7
Huang et al.	2019	China	Asian	M = NR F=NR	130 / 224	4.55 ± 3.19 / 5.02 ± 3.50	PCR–RFLP	7
Amar et al.	2019	Pakistani	Asian	M = NR F=NR	235 / 243	NR	PCR–RFLP	7
**TaqI (rs731236)**								
Jackman et al.	1999	USA	American	M = NR F=NR	17 / 37	NR	PCR–RFLP	5
Nishijima et al.	2002	Japan	Asian	M = NR F=NR	83 / 83	51.8 ± 15.6 / 54.4 ± 13.1	PCR–RFLP	5
Ozkaya et al.	2003	Turkey	European	M = 26/ 47 F = 38/43	64 / 90	6.7 ± 3.5 / 7.2 ± 2.3	PCR–RFLP	5
Mossetti et al.	2003	Italy	European	M = NR F=NR	220/114	40.87 ± 14.95 / 40.37 ± 14.07	PCR–RFLP	7
Shaogang et al.	2003	China	Asian	M = 89/ 58 F = 61/22	150 / 80	43.6 ± 16 / 49 ± 19.6	PCR–RFLP	6
Mossetti et al.	2004	Italy	European	M = 66/ 73 F = 44/54	110 / 127	41.3 ± 13.5 / 41.06 ± 13.9	PCR–RFLP	6
Gunes et al.	2006	Turkey	European	M = 67/ 73 F = 43/77	110 / 150	49.22 ± 1.33 / 48.15 ± 1.62	PCR–RFLP	7
Sayan et al.	2007	Turkey	European	M = 65/ 25 F = 15/15	80 / 40	10.9 ± 0.6 / 10.5 ± 0.6	PCR–RFLP	5
Moyano et al.	2007	Spain	European	M = 22/ 9 F = 29/12	51 / 21	45.5 ± 13.5 / 48.6 ± 15.4	PCR–RFLP	5
Seo et al.	2009	Korea	Asian	M = 93/ 220 F = 185/313	278 / 533	49.9 / 40.1	PCR–RFLP	8
Mittal et al.	2010	India	Asian	M = NR F=NR	125 / 150	40 ± 11.5 / 41.5 ± 10.5	PCR–RFLP	7
Basiri et al.	2012	Iran	Asian	M = NR F=NR	102 / 106	43.4 ± 6.9 / 38.4 ± 6.9	SSP-PCR	6
Wang et al.	2012	China	Asian	M = 279/263 F = 185/187	464 / 450	50.01 ± 10.19 / 50.45 ± 11.22	PCR–RFLP	8
Aykan et al.	2015	Turkey	European	M = 100/87 F = 64/ 80	164 / 167	24–58 / 38–54	PCR–RFLP	7
Guha et al.	2015	India	Asian	M = 133 / 112 F = 67 / 78	200 / 200	39.93 ± 11 / 38.13 ± 10	PCR	7
Rendina et al.	2016	Italy	European	M = NR F=NR	372 / 88	41.2 ± 13.3 / 40.8 ± 14.1	PCR–RFLP	7
Cakir et al.	2016	Turkey	European	M = 65 / 52 F = 33 / 18	98 / 70	47.2 ± 16.3 / 42.6 ± 13.5	PCR–RFLP	6
Goknar et al.	2016	Turkey	European	M = NR F=NR	78 / 60	6.94 ± 3.8 / 7.5 ± 3.2	PCR–RFLP	6
Subasi et al.	2017	Turkey	European	M = 26/22 F = 26/29	52 / 51	9.8 ± 3.5 / 10.3 ± 3.7	SNaPshot	5
Li et al.	2018	China	Asian	M = 100/60 F = 100/60	200 / 120	35.88 ± 14.2 / 36.16 ± 15.20	PCR	7
Yan*g* et al.	2019	China	Asian	M = 627/614 F = 316/361	943 / 975	51.2 ± 14.13 / 54.33 ± 18.11	iMLDR	8
Amar et al.	2019	Pakistani	Asian	M = NR F=NR	227 / 243	NR	PCR–RFLP	7
**BsmI (rs1544410)**								
Ruggiero et al.	1999	Italy	European	M = 18/NR F = 9/ NR	27 / 150	NR	PCR–RFLP	6
Chen et al. (a)	2001	China	Asian	M = 94/55 F = 30/ 35	124 / 90	44.1 ± 11.5 / 53 ± 10.1	PCR–RFLP	6
Ozkaya et al.	2003	turkey	European	M = 26/ 47 F = 38/43	64 / 90	6.7 ± 3.5 / 7.2 ± 2.3	PCR–RFLP	5
Rendina et al.	2004	Italy	European	M = 94/72 F = 65/52	159 / 124	43 ± 10.8 / 41.9 ± 10.4	PCR–RFLP	7
Mossetti et al.	2004	Italy	European	M = 66/ 73 F = 44/54	110 / 127	41.3 ± 13.5 / 41.06 ± 13.9	PCR–RFLP	6
Relan et al.	2004	India	Asia	M = 105/76 F = 45/24	150 / 100	39.3 ± 1.1 / 43.2 ± 2.05	PCR–RFLP	7
Gunes et al.	2006	turkey	European	M = 67/ 73 F = 43/77	110 / 150	49.22 ± 1.33 / 48.15 ± 1.62	PCR–RFLP	7
Moyano et al.	2007	Spain	European	M = 22/ 9 F = 29/12	51 / 21	45.5 ± 13.5 / 48.6 ± 15.4	PCR–RFLP	5
Wang et al.	2012	China	Asian	M = 279/263 F = 185/187	464 / 450	50.01 ± 10.19 / 50.45 ± 11.22	PCR–RFLP	8
Cakir et al.	2016	Turkey	European	M = 65 / 52 F = 33 / 18	98/ 70	47.2 ± 16.3 / 42.6 ± 13.5	PCR–RFLP	6
Goknar et al.	2016	Turkey	European	M = NR F=NR	72/ 53	6.94 ± 3.8 / 7.5 ± 3.2	PCR–RFLP	6
Subasi et al.	2017	turkey	European	M = 26/22 F = 26/29	52 / 51	9.8 ± 3.5 / 10.3 ± 3.7	SNaPshot	5
Li et al.	2018	China	Asian	M = 100/60 F = 100/60	200 / 120	35.88 ± 14.2 / 36.16 ± 15.20	PCR	7
Yang et al.	2019	China	Asian	M = 627/614 F = 316/361	943 / 975	51.2 ± 14.13 / 54.33 ± 18.11	iMLDR	8
**ApaI (rs7975232)**								
Nishijima et al.	2002	Japan	Asian	M = NR F=NR	83 / 83	51.8 ± 15.6 / 54.4 ± 13.1	PCR–RFLP	5
Shaogang et al.	2003	China	Asian	M = 89/ 58 F = 61/22	150 / 80	43.6 ± 16 / 49 ± 19.6	PCR–RFLP	6
Ozkaya et al.	2003	Turkey	Asian	M = 26/ 47 F = 38/43	64 / 90	6.7 ± 3.5 / 7.2 ± 2.3	PCR–RFLP	5
Rendina et al.	2004	Italy	European	M = 94/72 F = 65/52	159 / 124	43 ± 10.8 / 41.9 ± 10.4	PCR–RFLP	7
Gunes et al.	2006	Turkey	European	M = 67/ 73 F = 43/77	110 / 150	49.22 ± 1.33 / 48.15 ± 1.62	PCR–RFLP	7
Moyano et al.	2007	Spain	European	M = 22/ 9 F = 29/12	51 / 21	45.5 ± 13.5 / 48.6 ± 15.4	PCR–RFLP	5
Seo et al.	2009	Korea	Asian	M = 88/ 220 F = 185/305	273 / 525	49.9 / 40.1	PCR–RFLP	8
Mittal et al.	2010	India	Asian	M = NR F=NR	125 / 150	40 ± 11.5 / 41.5 ± 10.5	PCR–RFLP	7
Kaysar et al.	2012	China	Asian	M = NR F=NR	74 / 103	NR	PCR–RFLP	5
Wang et al.	2012	China	Asian	M = NR F=NR	463 / 450	50.01 ± 10.19 / 50.45 ± 11.22	PCR–RFLP	8
Cakir et al.	2016	Turkey	European	M = 65 / 52 F = 33 / 18	98/ 70	47.2 ± 16.3 / 42.6 ± 13.5	PCR–RFLP	6
Goknar et al.	2016	Turkey	European	M = NR F=NR	78/ 60	6.94 ± 3.8 / 7.5 ± 3.2	PCR–RFLP	6
Ergon et al.	2017	Turkey	European	M = NR F=NR	27 / 13	7.12 ± 2.64 / 6.92 ± 2.48	Tag-man	5
Subasi et al.	2017	Turkey	European	M = 26/22 F = 26/29	52 / 51	9.8 ± 3.5 / 10.3 ± 3.7	SNaPshot	5
Li et al.	2018	China	Asian	M = 100/60 F = 100/60	200 / 120	35.88 ± 14.2 / 36.16 ± 15.20	PCR	7
Yang et al.	2019	China	Asian	M = 627/614 F = 316/361	943 / 975	51.2 ± 14.13 / 54.33 ± 18.11	iMLDR	8

*Abbreviations*: *NR* not reported, *M* male, *F* female

**Table 2:** Distribution of genotype and allele among urolithiasis patients and controls

**Study author**	**Urolithiasis cases**					**Healthy control**					**P-HWE**	**MAF**
	**FF**	**Ff**	**ff**	**F**	**F**	**FF**	**Ff**	**ff**	**F**	**f**		
**FokI (rs2228570)**												
Chen et al. (b)	54	67	25	175	117	21	43	26	85	95	0/43	0/527
Shaogang et al.	27	64	59	118	182	17	44	19	78	82	0/36	0/512
Rendina et al.	69	68	22	206	112	53	55	16	161	87	0/77	0/350
Mossetti et al.	43	47	20	133	87	53	55	19	161	93	0/45	0/366
Relan et al.	25	72	53	122	178	38	36	26	112	88	0/01	0/44
Bid et al. (a)	30	106	2	136	90	77	84	5	238	94	0/02	0/257
Bid et al. (b)	11	38	1	60	40	30	28	2	88	32	0/13	0/266
Liu et al.	64	113	58	241	229	58	116	57	232	230	0/94	0/497
Seo et al.	84	134	60	302	254	155	288	92	598	472	0/03	0/441
Mittal et al.	25	98	2	214	86	69	76	5	148	102	< 0.01	0/408
Basiri et al.	54	42	6	150	54	36	27	43	99	113	< 0.01	0/533
Kaysar et al.	19	43	12	81	67	33	39	31	105	101	0/01	0/490
Wang et al.	150	234	80	534	394	125	226	99	476	424	0/86	0/471
Guha et al.	78	115	7	271	129	98	90	12	286	114	0/74	0/542
Cakir et al.	48	38	12	134	62	39	25	6	103	37	0/39	0/618
Ergon et al.	14	12	1	40	14	7	6	0	20	6	0/27	0/230
Subasi et al.	23	25	4	71	33	26	21	4	73	29	0/93	0/284
Li et al.	38	102	60	178	222	31	72	17	134	106	0/02	0/4416
Huang et al.	73	49	8	195	65	104	96	24	304	144	0/79	0/321
Amar et al.	136	79	11	351	101	146	77	10	369	97	0.37	0.519
**Study author**	**Urolithiasis cases**					**Healthy control**					**P-HWE**	**MAF**
	**TT**	**Tt**	**tt**	**T**	**t**	**TT**	**Tt**	**tt**	**T**	**t**		
**TaqI (rs731236)**												
Jackman et al.	6	7	4	19	15	17	8	12	42	32	0/82	0/432
Nishijima et al.	49	30	4	128	38	60	22	1	142	24	0/1	0/228
Ozkaya et al.	33	27	4	93	35	50	30	10	130	50	0/81	0/277
Mossetti et al.	80	104	36	264	176	35	66	13	136	92	0/53	0/719
Shaogang et al.	52	74	24	178	122	33	36	11	102	58	0/3	0/362
Mossetti et al.	21	53	36	95	125	21	68	38	110	144	0/39	0/566
Gunes et al.	37	63	10	137	83	61	73	16	195	105	0/02	0/35
Shayan et al.	27	35	18	89	71	13	25	2	51	29	0/74	0/362
Moyano et al.	15	23	13	53	49	9	10	2	28	14	< 0.01	0/333
Seo et al.	252	23	3	527	29	487	39	7	1013	53	0/05	0/049
Mittal et al.	56	61	8	173	77	84	50	16	218	82	0/03	0/273
Basiri et al.	41	50	11	132	72	52	37	17	141	71	0/77	0/334
Wang et al.	430	32	2	892	36	414	35	1	863	37	0/08	0/041
Aykan et al.	67	61	36	195	133	66	86	15	218	116	< 0.01	0/347
Guha et al.	58	82	60	196	202	65	58	77	188	212	0/67	0/349
Rendina et al.	186	158	28	530	214	31	44	13	106	70	0/16	0/473
Cakir et al.	35	44	19	114	82	31	29	10	91	49	0/43	0/173
Goknar et al.	25	41	12	91	65	14	43	3	71	49	0/83	0/408
Subasi et al.	4	25	23	33	71	9	24	18	42	60	0/77	0/588
Li et al.	189	11	0	389	11	114	6	0	234	6	0/82	0/025
Yang et al.	849	92	2	1790	96	870	103	2	1843	107	0/67	0/471
Amar et al.	112	86	29	310	144	116	104	23	336	150	0/42	0/149
**Study author**	**Urolithiasis cases**					**Healthy control**					**P-HWE**	**MAF**
	**BB**	**Bb**	**bb**	**B**	**b**	**BB**	**Bb**	**bb**	**B**	**b**		
**BsmI (rs1544410)**												
Ruggiero et al.	4	12	11	19	35	18	108	24	144	156	< 0.01	0/52
Chen et al. (a)	110	10	4	230	18	78	9	3	165	15	< 0.01	0/083
Ozkaya et al.	5	36	23	46	82	13	49	28	75	105	0/25	0/583
Rendina et al.	47	69	43	163	155	39	56	29	134	114	0/31	0/459
Mossetti et al.	40	46	24	126	94	40	56	31	136	118	0/2	0/464
Relan et al.	48	62	40	158	142	46	28	26	120	80	< 0.01	0/40
Gunes et al.	15	64	31	94	126	19	75	56	113	187	0/42	0/623
Moyano et al.	5	25	21	35	67	5	9	7	19	23	0/53	0/547
Wang et al.	3	66	395	72	856	2	70	378	74	826	0/51	0/917
Cakir et al.	43	40	15	126	70	26	34	10	86	54	0/57	0/476
Goknar et al.	21	35	16	77	67	16	37	0	69	37	0/01	0/349
Subasi et al.	28	19	5	75	29	20	23	8	63	39	0/74	0/382
Li et al.	181	19	0	381	19	111	9	0	231	9	0/67	0/0375
Yang et al.	65	394	484	524	1362	78	417	480	573	1377	0/28	0/315
**Study author**	**Urolithiasis cases**					**Healthy control**					**P-HWE**	**MAF**
	**AA**	**Aa**	**aa**	**A**	**a**	**AA**	**Aa**	**aa**	**A**	**a**		
**ApaI (rs7975232)**												
Nishijima et al.	14	34	35	62	104	9	37	37	55	111	0/25	0/626
Shaogang et al.	32	69	49	133	167	11	38	31	60	100	0/9	0/625
Ozkaya et al.	13	30	21	56	72	4	50	36	58	122	0/09	0/677
Rendina et al.	43	87	29	173	145	37	68	19	142	106	0/18	0/427
Gunes et al.	40	58	12	138	82	59	72	19	190	110	0/68	0/366
Moyano et al.	11	29	11	51	51	7	9	5	23	19	0/53	0/452
Seo et al.	152	84	37	388	158	282	192	51	756	294	0/03	0/28
Mittal et al.	43	70	12	156	94	57	71	22	185	115	0/98	0/383
Kaysar et al.*.*	21	29	24	71	77	32	42	29	106	100	0/06	0/485
Wang et al	27	177	259	231	695	46	195	209	287	613	0/75	0/748
Cakir et al.	43	40	15	126	70	26	34	10	86	54	0/63	0/135
Goknar et al.	24	42	12	90	66	11	40	9	62	58	0/01	0/483
Ergon et al.	9	12	6	30	24	4	6	3	14	12	0/79	0/461
Subasi et al.	18	24	10	60	44	22	14	15	58	44	0/01	0/431
Li et al.	73	87	40	233	167	57	51	12	165	75	0/9	0/312
Yang et al.	65	394	484	524	1362	78	417	480	573	1377	0/49	0/743

*Abbreviations*: *P-HWE p*-value for Hardy–Weinberg equilibrium, *MAF* minor allele frequency of control group

### Quantitative synthesis

As the reference categories for comparing, the FF genotype for FokI SNP, TT genotype for TaqI SNP, BB genotype for BsmI SNP, and AA genotype for ApaI were used.

### Association of FokI polymorphism and urolithiasis risk

Twenty studies, including 3114 urolithiasis patients and 3174 controls, evaluated the FokI polymorphism. Of which, 14 studies were conducted in Asian countries [26, 28, 34, 35, 37, 40–42, 44, 50, 60–63] and 6 studies were in European countries [31–33, 48, 64, 65]. Overall, no significant association was detected between *FokI* SNP and urolithiasis risk under all five genetic models. Besides, the findings of subgroup analysis reject any association between *FokI* SNP and risk of urolithiasis in East-Asians and Caucasians.

### Association of TaqI polymorphism and urolithiasis risk

**Fig. 2:**
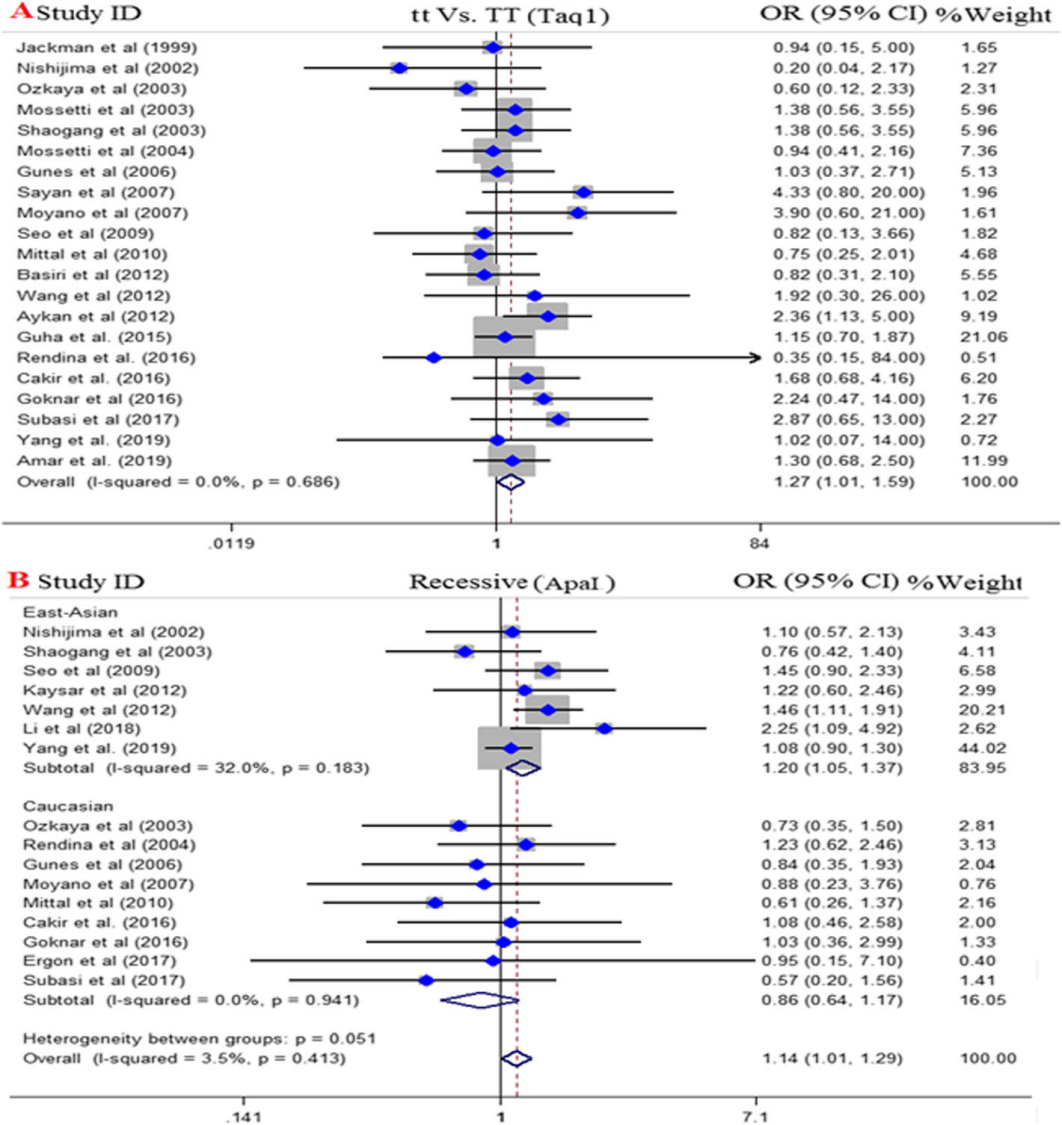
Pooled odds ratio (OR) and 95% confidence interval of individual studies and pooled data for the association between *TaqI* and *ApaI* gene polymorphism and urolithiasis risk in different ethnicity subgroups and overall populations. **a**; tt vs. TT Model (*TaqI*) and **b**: A; Recessive Model (*ApaI*)

Twenty-two case-control studies with 4188 cases and 3955 controls met inclusion criteria for evaluating the association between TaqI SNP and urolithiasis risk. Among them, ten studies were performed in Asian population [28, 40–42, 44, 60–63, 66] and eleven studies were in European population [29, 31, 36, 38, 39, 45, 46, 48, 65, 67] and one study in the USA [24]. The pooled results did not indicate significant association between TaqI SNP and urolithiasis risk except in tt vs. TT model (OR = 1.27, 95% CI = 1.01–1.59, *P* = 0.04), also subgroup analysis revealed that the tt genotype was associated with urolithiasis risk in Caucasians when compared with the TT genotype [tt vs. TT (OR = 1.30, 95% CI = 1.021–1.65, *P* = 0.03)], Fig. 2.

### Association of BsmI polymorphism and urolithiasis risk

Fourteen eligible publications with 3065 cases and 2915 controls were included and evaluated the association between BsmI polymorphism and urolithiasis risk. Among 14 studies, only five publications were carried out in Asia [27, 44, 62, 66, 68] and nine studies were in Europe [25, 29, 31–33, 36, 38, 46, 48, 65]. The statistical analysis demonstrated that there was no significant association between *BsmI* SNP and urolithiasis risk under any genetic models in both the overall population and the subgroup analysis.

### Association of ApaI polymorphism and urolithiasis risk

A total of 16 publications containing 2950 cases and 3065 controls were recognized eligible for evaluating the association between ApaI SNP and urolithiasis risk. Of which, eight studies were performed in Asians [27, 28, 40, 44, 61, 66, 68] and eight studies were in Europeans [29, 33, 36, 38, 41, 45, 46, 48, 64]. The pooled results revealed a marginal significant association between *ApaI* SNP and urolithiasis risk under recessive model (OR = 1.14, 95% CI = 1.01–1.29, *p* = 0.03), allelic model (OR = 1.09, 95% CI = 1–1.18, *P* = 0.05), and aa vs. AA model (OR = 1.21, 95% CI = 1–1.47, *P* = 0.05). Additionally, the results of subgroup analysis indicated a positive significant association in East-Asians across recessive model (OR = 1.20, 95% CI = 1.05–1.37, *P* < 0.001), allelic model (OR = 1.15, 95% CI = 1.05–1.26, *P* < 0.001), and aa vs. AA model (OR = 1.40, 95% CI = 1.12–1.75, *P* < 0.001) but not Caucasians. The results of pooled ORs, heterogeneity tests, and publication bias tests for different analysis models are shown in Table 3.

**Table 3:** Main results of pooled ORs in meta-analysis of *VDR* gene polymorphisms

Subgroup		Sample size	Test of association		Test of heterogeneity		Test of publication bias (Begg’s test)		Test of publication bias (Egger’s test)	
	Genetic model	Case/Control	OR	95%CI (***p***-value)	I^**2**^ (%)	P	Z	P	T	P
**FokI (rs2228570)**										
**Overall**	Dominant model	3114 / 3174	1.16	0.90–1.50(0.25)	77.7	≤0.001	1.73	0.08	1.37	0.19
	Recessive model	3114 / 3174	0.92	0.68–1.25(0.58)	67.2	≤0.001	−1.17	0.24	−0.68	0.50
	Allelic model	3114 / 3174	1.02	0.86–1.22(0.82)	78.5	≤0.001	0.25	0.80	0.45	0.66
	ff vs. FF	3114 / 3174	1.10	0.72–1.69(0.65)	77.9	≤0.001	−0.72	0.47	−0.14	0.88
	Ff vs. FF	3114 / 3174	1.12	0.88–1.43(0.34)	74.1	≤0.001	1.94	0.05	1.39	0.18
**East-Asian**	Dominant model	1677 / 1833	0.91	0.77–1.06(0.22)	1	0.42	0.99	0.32	1.64	0.15
	Recessive model	1677 / 1833	0.98	0.66–1.45(0.91)	74.9	≤0.001	0	1	−0.18	0.86
	Allelic model	1677 / 1833	0.95	0.78–1.16(0.62)	71.6	≤0.001	−0.25	0.80	0.15	0.88
	ff vs. FF	1677 / 1833	0.93	0.61–1.40(0.71)	67.7	≤0.001	0	1	0.10	0.92
	Ff vs. FF	1677 / 1833	0.88	0.74–1.04(0.12)	0	0.48	0.78	0.45	0.84	0.43
**Caucasian**	Dominant model	1437 / 1341	1.33	0.87–2.05(0.18)	83.6	≤0.001	1.04	0.29	0.62	0.55
	Recessive model	1437 / 1341	0.84	0.49–1.44(0.52)	62.8	≤0.001	−1.73	0.08	−0.69	0.51
	Allelic model	1437 / 1341	1.08	0.81–1.45(0.59)	82.1	≤0.001	−0.21	0.83	0.2	0.82
	ff vs. FF	1437 / 1341	1.28	0.56–2.94(0.55)	81	≤0.001	−0.25	0.80	−0.68	0.52
	Ff vs. FF	1437 / 1341	1.33	0.90–1.98(0.15)	75.5	≤0.001	1.25	0.21	0.56	0.59
**TaqI (rs731236)**										
**Overall**	Dominant model	4188 / 3955	1.05	0.93–1.19(0.41)	14	0.27	0.45	0.65	0.40	0.69
	Recessive model	4188 / 3955	1.07	0.88–1.30(0.48)	31.5	0.08	0.05	0.96	−0.23	0.83
	Allelic model	4188 / 3955	1.06	0.97–1.16(0.23)	2.6	0.42	−0.18	0.85	−0.21	0. 83
	tt vs. TT	4188 / 3955	**1.27**	**1.01–1.59(0.04)**	0	0.68	0.05	0.96	−0.06	0.95
	Tt vs. TT	4188 / 3955	1.04	0.91–1.18(0.59)	34.5	0.05	0.45	0.65	0.33	0.74
**East-Asian**	Dominant model	2118/ 2241	0.94	0.76–1.16(0.55)	0	0.56	0.19	0.85	−0.19	0.85
	Recessive model	2118/ 2241	0.97	0.50–1.88(0.92)	0	0.68	−0.49	0.62	−1.45	0.24
	Allelic model	2118/ 2241	0.95	0.79–1.15(0.60)	0	0.51	−1.69	0.09	−1.27	0.27
	tt vs. TT	2118/ 2241	1.02	0.51–2.02(0.96)	0	0.50	−0.98	0.32	−1.70	0.18
	Tt vs. TT	2118/ 2241	0.94	0.76–1.16(0.26)	0	0.67	0.56	0.57	0.37	0.73
**Caucasian**	Dominant model	2070 / 1714	1.12	0.96–1.29(0.15)	20.3	0.22	0.09	0.92	−0.03	0.97
	Recessive model	2070 / 1714	1.08	0.88–1.33(0.44)	44.1	0.03	1.16	0.24	0.92	0.38
	Allelic model	2070 / 1714	1.09	0.98–1.21(0.09)	4.5	0.40	1.34	0.18	1.58	0.15
	tt vs. TT	2070 / 1714	**1.30**	**1.02–1.65(0.03)**	0	0.62	1.52	0.12	2.35	0.04
	Tt vs. TT	2070 / 1714	1.10	0.93–1.29(0.56)	45.7	0.02	−0.80	0.42	−0.41	0.69
**BsmI (rs1544410)**										
**Overall**	Dominant model	3065/2915	0.97	0.84–1.12(0.69)	12	0.31	0.41	0.68	- 0.04	0.96
	Recessive model	3065/2915	0.98	0.86–1.12(0.74)	38.7	0.06	0.27	0.78	0.46	0.65
	Allelic model	3065/2915	0.99	0.91–1.08(0.82)	42.5	0.03	0.55	0.58	0.74	0.47
	bb vs. BB	3065/2915	0.95	0.79–1.14(0.56)	22.2	0.21	0.27	0.78	0.10	0.92
	Bb vs. BB	3065/2915	0.97	0.83–1.14(0.74)	0.8	0.44	0	1	0.38	0.71
**East-Asian**	Dominant model	1783 / 1686	0.86	0.71–1.05(0.41)	0	0.76	0.52	0.60	−0.47	0.72
	Recessive model	1783 / 1686	0.88	0.73–1.05(0.16)	0	0.59	−1.00	0.31	–	–
	Allelic model	1783 / 1686	0.89	0.79–1.01(0.06)	0	0.58	0.52	0.60	−0.04	0.97
	bb vs. BB	1783 / 1686	0.78	0.60–1.00(0.05)	0	0.88	−1.00	0.31	–	–
	Bb vs. BB	1783 / 1686	0.91	0.73–1.12(0.36)	0	0.81	−0.52	0.60	0.87	0.54
**Caucasian**	Dominant model	1282/ 1229	1.11	0.90–1.36(0.34)	18.6	0.26	0	1	0.22	0.83
	Recessive model	1282/ 1229	1.11	0.91–1.35(0.30)	45.3	0.05	0.49	0.62	0.81	0.45
	Allelic model	1282/ 1229	1.10	0.97–1.24(0.12)	43.2	0.06	0.83	0.40	0.92	0.38
	bb vs. BB	1282/ 1229	1.16	0.89–1.50(0.26)	21.5	0.24	0.99	0.32	0.47	0.65
	Bb vs. BB	1282/ 1229	1.06	0.84–1.32(0.63)	20.7	0.24	- 0. 21	0.83	- 0.09	0.93
**ApaI (rs7975232)**										
**Overall**	Dominant model	2950 / 3065	1.08	0.93–1.25(0.30)	48.6	0.01	- 0.35	0.72	- 0.54	0.60
	Recessive model	2950 / 3065	**1.14**	**1.01–1.29(0.03)**	3.5	0.41	- 0.38	0.70	- 0.02	0.98
	Allelic model	2950 / 3065	**1.09**	**1.00–1.18(0.05)**	31	0.11	- 0.64	0.52	0..42	0.67
	aa vs. AA	2950 / 3065	**1.21**	**1.00–1.47(0.05)**	27.5	0.14	- 1.15	0.25	- 0.85	0.41
	Aa vs. AA	2950 / 3065	1.10	0.94–1.28(0.29)	41.1	0.04	- 0.94	0.34	- 0.39	0.70
**East-Asian**	Dominant model	2186/ 2336	1.15	0.96–1.38(0.12)	38.8	0.13	0.19	0.85	0.21	0.84
	Recessive model	2186/ 2336	**1.20**	**1.05–1.37(≤0.001)**	32	0.18	1.69	0.09	1.37	0.24
	Allelic model	2186/ 2336	**1.15**	**1.05–1.26(≤0.001)**	49.1	0.06	0.19	0.85	- 0.09	0.93
	aa vs. AA	2186/ 2336	**1.40**	**1.12–1.75(≤0.001)**	36.1	0.15	- 0.19	0.85	0.45	0.67
	Aa vs. AA	2186/ 2336	1.10	0.90–1.33(0.35)	40.7	0.12	0.56	0.57	0.54	0.62
**Caucasian**	Dominant model	764 / 729	0.96	0.75–1.22(0.73)	55.4	0.02	- 0.83	0.40	- 1.14	0.29
	Recessive model	764 / 729	0.86	0.64–1.17(0.34)	0	0.94	0.49	0.62	- 0.25	0.81
	Allelic model	764 / 729	0.94	0.80–1.09(0.40)	0	0.75	0.42	0.67	0.60	0.56
	aa vs. AA	764 / 729	0.83	0.58–1.20(0.32)	0	0.68	- 0.99	0.32	- 1.32	0.23
	Aa vs. AA	764 / 729	1.10	0.85–1.42(0.45)	47.9	0.05	- 1.25	0.21	- 1.40	0.20

### Evaluation of the heterogeneity and publication bias

**Fig. 3:**
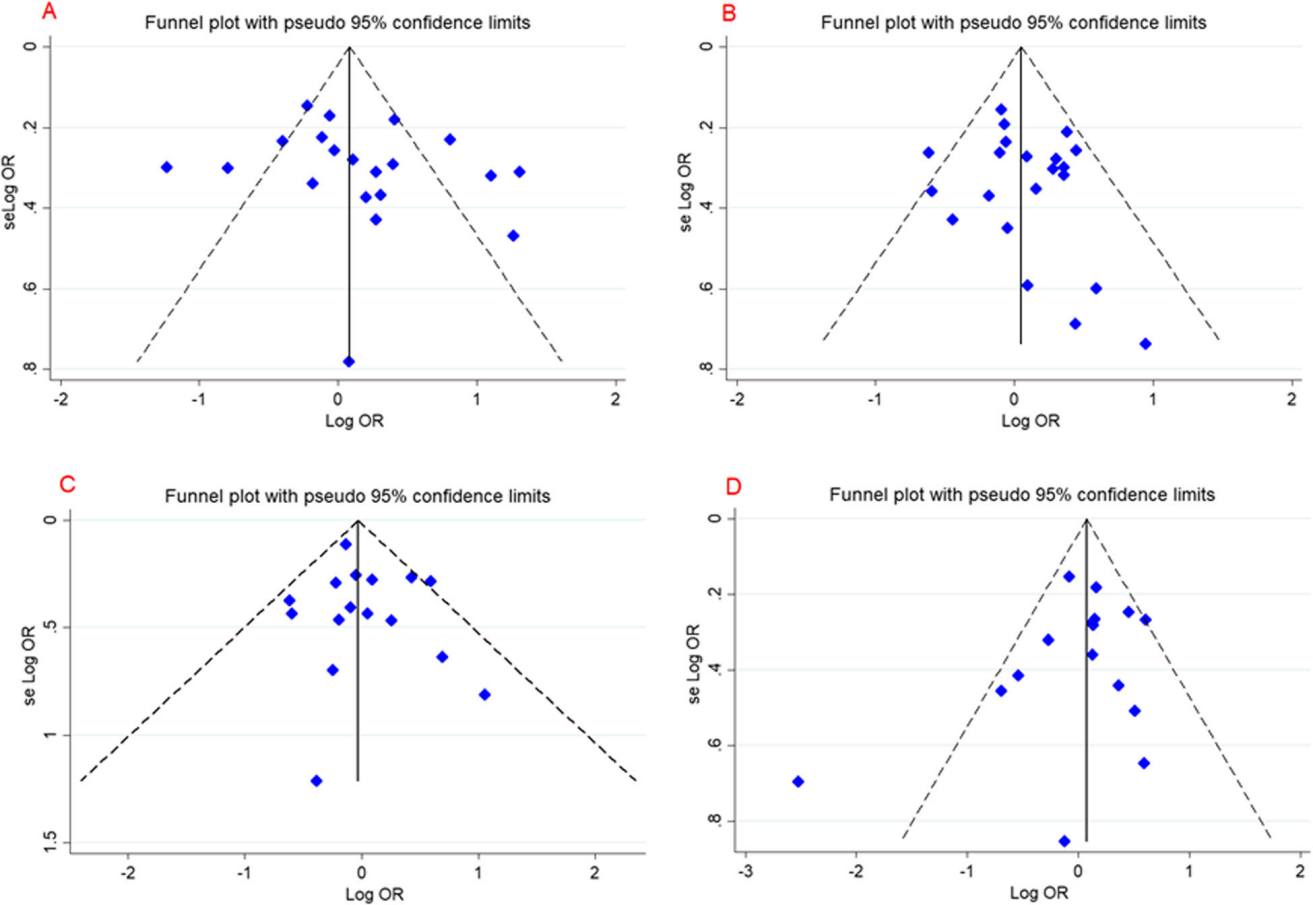
Begg’s funnel plot for publication bias test. **a**; Dominant Model *FokI*, **b**; Dominant Model *TaqI*, **c**; Dominant Model *BsmI*, **d**; Dominant Model *ApaI*. Each point represents a separate study for the indicated association

The results of publication bias test indicated that there was no evidence of publication bias for overall population and subgroup analysis of all FokI, TaqI, BsmI, and ApaI SNPs. Additionally, the shape of the funnel plot confirmed absence of publication bias. No heterogeneity in both the overall and subgroup analyses was detected except for FokI polymorphism (Fig. 3, Table 3).

### Sensitivity analysis

**Fig. 4:**
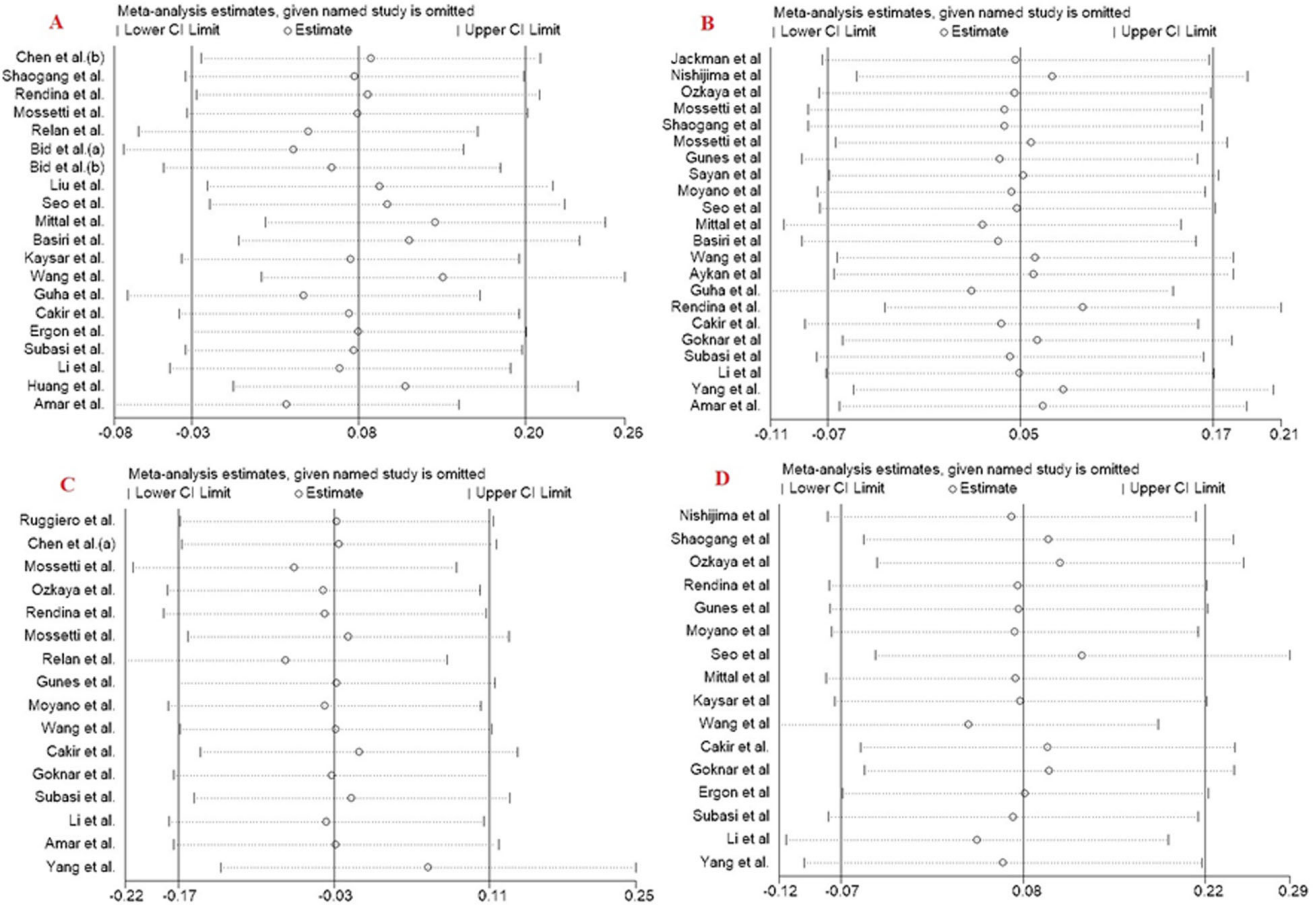
Sensitivity analysis in present meta-analysis investigates the single nucleotide polymorphisms of Vitamin D Receptor contribute to risk for urolithiasis susceptibility (**a**, *FokI*; **b**, *TaqI*; **c**, *BsmI*; **d**, *ApaI*)

Sensitivity analysis is an effective test to evaluate the influence of individual study on the pooled results. In the sensitivity analysis, each eligible study was sequentially removed to assess whether the individual data influence the pooled ORs. In this meta-analysis, the pooled results did not significantly affect by any single study in the dominant model for FokI, TaqI, BsmI and ApaI SNPs (Fig. 4), indicating that the combined results of our meta-analysis were statistically robust.

### Meta-regression analyses

**Fig. 5:**
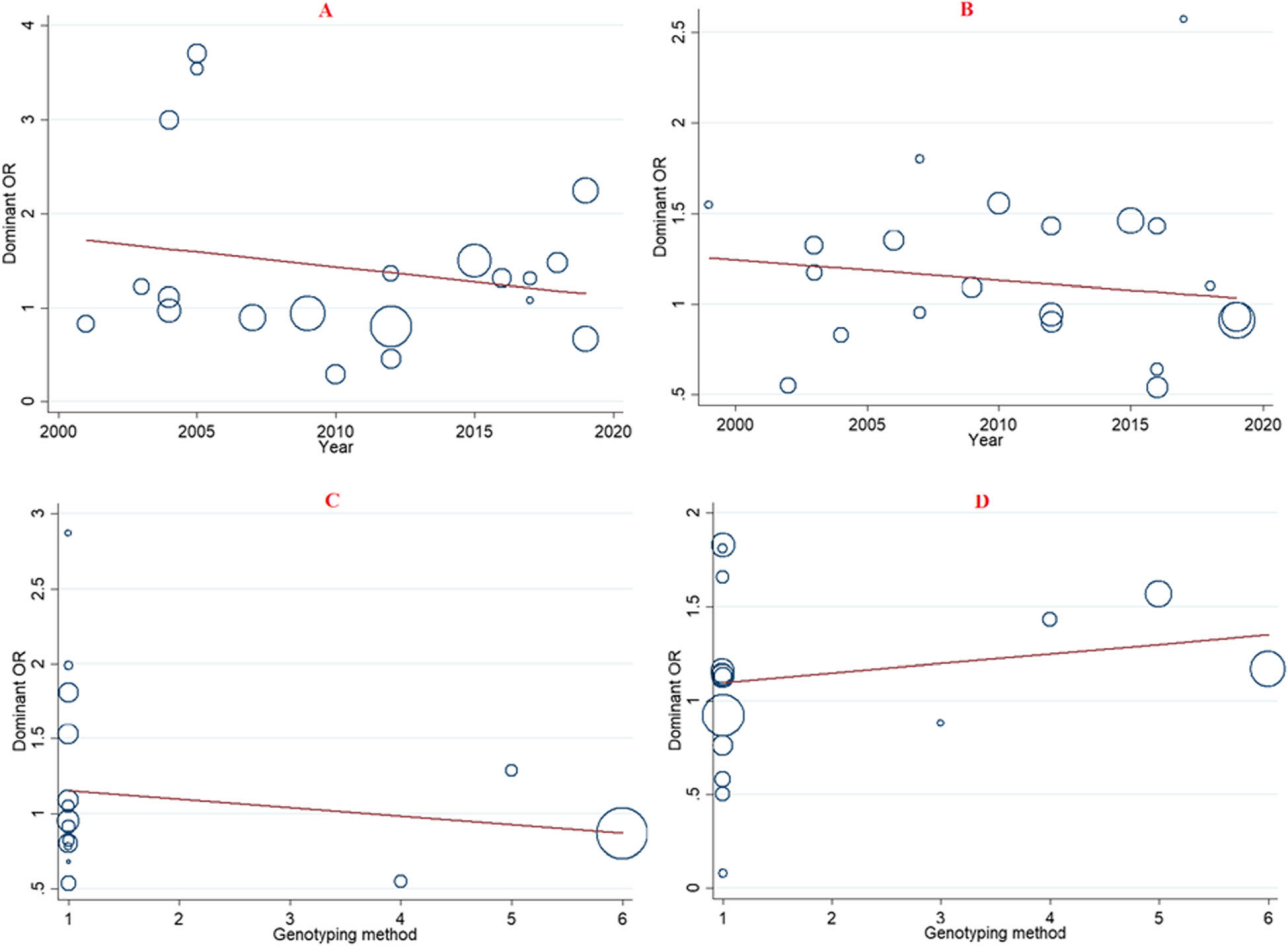
Meta-regression plots of the association between VDR gene polymorphisms and risk of urolithiasis (Dominant model) based on; **a**: Publication year (FokI), **b**: Publication year (TaqI), **c**: Genotyping method (BsmI), **d**: Genotyping method (ApaI)

Potential sources of heterogeneity among included studies was estimated by meta-regression analyses (Table 4). According that, the findings indicated that none of the expected heterogeneity parameter were the source of heterogeneity for the association between VDR gene polymorphism and the risk of urolithiasis (Fig. 5).

**Table 4:** Meta-regression analyses of potential source of heterogeneity

Heterogeneity Factor		Coefficient	SE	T-test	***P***-value	95% CI	
						UL	LL
**FokI**							
**Dominant**	Publication Year	−0.031	0.03	−0.87	0.39	−0.108	0.045
	Genotyping Method	−0.032	0.16	−0.20	0.84	−0.370	0.306
**TaqI**							
**Dominant**	Publication Year	−0.011	0.01	−0.86	0.40	−0.037	0.015
	Genotyping Method	0.018	0.04	0.42	0.67	−0.073	0.109
**BsmI**							
**Dominant**	Publication Year	−0.025	0.013	−1.93	0.07	−0.054	0.002
	Genotyping Method	−0.056	0.58	−0.97	0.34	−0.181	0.068
**ApaI**							
**Dominant**	Publication Year	0.012	0.018	0.68	0.50	−0.026	0.051
	Genotyping Method	0.050	0.051	0.99	0.34	−0.059	0.160

## Discussion

In the current most recent meta-analysis, 33 case-control association studies evaluating the *VDR* gene SNPs and urolithiasis risk were analyzed. The results of pooled analysis revealed that none of the four SNPs in *VDR* gene were in significant association with proneness to urolithiasis. That notwithstanding, subgroup analysis based on the population stratification demonstrated increased risk of urolithiasis in East-Asian (recessive, allelic and aa vs. AA model) and Caucasian (heterozygous model) population with ApaI and *TaqI* gene polymorphism, respectively.

Several investigations have noted that *VDR* gene SNPs have been contributing genetic factors in susceptibility to urolithiasis [27, 31, 34, 49]. A bulk of studies have attempted to disclose the possible association between *VDR* gene SNPs and urolithiasis risk; that notwithstanding, the findings still show discrepancies and a comprehensive meta-analysis seems to be required to shed insights on the unknown conundrums. As a result, we performed a meta-analysis to investigate the consequence of the common four SNPs in the VDR gene, namely FokI (rs2228570), TaqI (rs731236), BsmI (rs1544410), and ApaI (rs7975232) on the risk of urolithiasis. The discrepancies in outcome among the various ethnicities might be due to differences in geographic and ethnical diversity, and impression of ethnicity on the serum level of vitamin D as well as the *VDR* gene expression [69].

Reports have shown the role of environmental factors on the risk of different diseases. For example, seasonal differences may impress the serum level of vitamin D [70]. Among the pregnant women in south-eastern USA, season was indicated to be associated with vitamin D levels in non-Hispanic women [71]. Sun exposure has been shown to interact with functional variants of the *VDR* gene [72]. Additionally, sun exposure and the differences between high and low latitudes, it has been implied that people in high latitude regions experience lower levels of vitamin D, especially in those with darker skin (which is a natural barrier to the UV radiation) [73]. As a result, environmental stimuli may impress the functional variants of the *VDR* gene as well as serum levels of vitamin D and, hence, modify the risk of urolithiasis susceptibility, along with VDR genetic polymorphisms.

Vitamin D is a critical hormone and play a role in the metabolism of calcium. This vitamin implements its function by binding to the VDR. The genetic variations in the *VDR* gene have been shown to impress the interactions of the vitamin D/VDR, modulating the susceptibility risk for several pathologic conditions. FokI polymorphism can modulate the ATG start cordon in the VDR protein and BsmI SNP can modify the VDR protein expression [74, 75]. Additionally, ApaI and TaqI SNPs have been shown to have potential to modify the mRNA transcription of *VDR* gene and can modulate the stability of VDR mRNA [21]. FokI SNP has been shown to have potential to modulate the function of transcription factors [76, 77].

A recent meta-analysis by González-Castro in 2019 [78], including 23 studies (a total of 1536 cases/1767 controls for ApaI polymorphism, 1571 cases/ 1455 controls for BsmI polymorphism, 2145 cases/2280 controls for FokI polymorphism, and 2160 cases/2307 controls for TaqI polymorphism), indicated that BsmI polymorphism had a protective association with nephrolithiasis in the allelic and homozygous models. Moreover, both TaqI polymorphism and FokI polymorphism were associated with a decreased risk of nephrolithiasis in the heterozygous model. However, no association of ApaI polymorphism was detected with nephrolithiasis. However, our most recent update meta-analysis in 2020, by including 33 studies (a total of 2950 cases/3065 controls for ApaI polymorphism, 3065 cases/ 2915 controls for BsmI polymorphism, 3114 cases/3174 controls for FokI polymorphism, and 4188 cases/3955 controls for TaqI polymorphism), indicated that none of the *VDR* gene polymorphisms mentioned above were associated significantly with nephrolithiasis risk in the overall analysis except ApaI SNP. However, our subgroup analysis according to population stratification revealed that ApaI gene polymorphism increased risk of urolithiasis in East-Asian patients by the recessive, allelic and homozygous model and TaqI gene SNP in Caucasians population through the heterozygous model. On the other hand, a meta-analysis in 2014 with respect to the study of the associations between *VDR* gene SNPs and urolithiasis risk included 20 studies in the analysis [53]. They found that the TaqI polymorphism was associated with an increased risk of urolithiasis, whereas the ApaI, BsmI, and FokI polymorphisms did not show any significant association. Moreover, stratifying for ethnicity, a slightly increased risk was found among Asians as compared with Whites for TaqI SNP. On the other hand, our meta-analysis on 33 studies did not result in any strong significant association between all four SNPs and urolithiasis risk in the pooled overall comparison. However, subgroup analysis demonstrated a significant increased risk of urolithiasis in East-Asian and Caucasians populations in association with ApaI and TaqI genes polymorphism. In the current meta-analysis, thirteen more studies were added in comparison to the previous study, and did not support the previous finding in the overall analysis.

The subgroup analyses were conducted based on the ethnicity to identify the potential impression of the genetic background on the association of VDR gene polymorphisms and urolithiasis. Our analysis resulted in identification of ApaI and TaqI polymorphism association with increased risk of urolithiasis in East-Asian and Caucasians populations. However, the previous meta-analysis identified the same association in only Asians [53]. These discrepancies may stem from diversities in the genetic backgrounds. Furthermore, given that solar UV radiation is involved in the process of vitamin D generation [79], the significant association of *VDR* gene TaqI SNP in Asians might be attributed to the partially higher amount of exposure to UVR [80]. Moreover, it has been implied that level of UV exposure may impress that the associations between *VDR* gene polymorphisms and disorders. In patients with non-Hodgkin lymphoma, it was reported that patients with CC genotype for TaqI SNP who experienced sun exposure less than 7 h per week exhibited higher risk of the disease in comparison to patients with TT genotype with the similar duration of sun exposure [81]. In addition, reports showed that the TaqI T allele was more common in prostate cancer patients in a southern European population compared with the controls [82]. Plus, in a British population, the association of FokI polymorphism was observed to be limited to cases with a high exposed to UV [83]. Other than that, gender has been known as also a major risk factor for urolithiasis risk. It was shown that the FokI polymorphism had significant differences in females but not males, implying to the role of gender on the function of VDR [44]. Nonetheless, lack of sufficient data hindered the subgroup analysis based on gender in the current meta-analysis, which need to be addressed in the further studies.

Data from GWASs as well as association studies in different ethnic groups have revealed that *VDR* gene polymorphisms play a role in altering the risk of urolithiasis development. Although our analysis did not endorse the association of *VDR* gene BsmI, ApaI, FokI, and TaqI SNPs with susceptibility to urolithiasis, the gene can be of beneficial applications in populations with significant associations. Generally, the concept of personalized medicine has been widely accepted, implying to the consideration of genetic makeup of each patient in approaching with optimized medication. As a consequence, clarification of VDR gene polymorphisms contribution to the urolithiasis predisposition could be advantageous in clinics with respect to better diagnosis of subjects at risk as well as treatment with maximum efficacy.

Despite we tried to perform the possibly well-suited analysis of the available data, a number of caveats and confining factors are related to this meta-analysis. First, our literature search was limited to only English-written papers, raising the chance of excluding of potentially worthwhile findings. Second, we could not analyze the role of age, gender, lifestyle, and other genetic variations, on the adjusted association of *VDR* gene SNPs and urolithiasis risk. Hence, additional works with respect to the gene–gene and gene–environment interactions is needed to approach with a more comprehensive estimation. Third, we noticed a significant heterogeneity among the studies for various comparisons, which may impress the perception of findings. Although we conducted subgroup analysis and weighted meta-regression in order to attenuate its effects. Finally, there were a number of *VDR* gene SNPs in the context of urolithiasis risk that could not be included in the meta-analysis due to lack of sufficient amount of data. Hence, it could barely implied that *VDR* gene could not convey a genetic risk factor for urolithiasis, merely regarding our findings.

## Conclusion

In conclusion, the results of pooled analysis did not demonstrate any statistically significant association between all four SNPs and susceptibility to urolithiasis. However, subgroup analysis showed that the Recessive, allelic, and aa vs. AA model of ApaI and Tt vs. TT comparison of the *TaqI* gene polymorphism increased risk of urolithiasis in East-Asian and Caucasians population, respectively. Further genes should be evaluated to disclose the genetic mechanisms contributing to urolithiasis development. Moreover, the role of life style, age, and gender needs be considered in the stratification analyses for *VDR* gene SNPs and urolithiasis predisposition.

## Data Availability

Not applicable.

## Abbreviations

VD: Vitamin D receptor
CI: Confidence interval
OR: Odds ratio
SNP: Single-nucleotide polymorphism
CaSR: Calcium sensing receptor
OPN: Osteopontin
PAQR6: Progestin and adipo receptor 6
PRISMA: Preferred Reporting Items for Systematic reviews and Meta-Analyses
NOS: Newcastle–Ottawa scale
HWE: Hardy–Weinberg equilibrium
RXR: Retinoid X receptor
VDRE: Vitamin D response element
